# Studies on the Role of Compartmentalized Profiles of Cytokines in the Risk of Hepatocellular Carcinoma

**DOI:** 10.3390/ijms241713432

**Published:** 2023-08-30

**Authors:** Silvano Fasolato, Paola Del Bianco, Sandro Malacrida, Adriana Mattiolo, Enrico Gringeri, Paolo Angeli, Patrizia Pontisso, Maria Luisa Calabrò

**Affiliations:** 1 Department of Medicine, Padua University Hospital, I-35128 Padua, Italy; pangeli@unipd.it (P.A.); patrizia@unipd.it (P.P.); 2 Clinical Research Unit, Veneto Institute of Oncology IOV-IRCCS, I-35128 Padua, Italy; paola.delbianco@iov.veneto.it; 3 Institute of Mountain Emergency Medicine, Eurac Research, I-39100 Bozen, Italy; sandro.malacrida@eurac.edu; 4 Immunology and Molecular Oncology, Veneto Institute of Oncology IOV-IRCCS, I-35128 Padua, Italy; adriana.mattiolo86@gmail.com (A.M.); luisella.calabro@iov.veneto.it (M.L.C.); 5 Hepatobiliary Surgery and Liver Transplantation, Padua University Hospital, I-35128 Padua, Italy; enrico.gringeri@unipd.it

**Keywords:** hepatocellular carcinoma, cytokines, biomarker, inflammation, combination therapy, immunotherapy

## Abstract

Hepatocellular carcinoma (HCC), the most common form of liver cancer, is frequently diagnosed late due to the absence of symptoms during early disease, thus heavily affecting the overall survival of these patients. Soluble immunological factors persistently produced during cirrhosis have been recognized as promoters of chronic inflammation and neoplastic transformation. The aim of this pilot study was to evaluate the predictive value of the cytokine profiles for HCC development. A Luminex xMAP approach was used for the quantification of 45 proteins in plasma and ascitic fluids of 44 cirrhotic patients without or with HCC of different etiologies. The association with patient survival was also evaluated. Univariate analyses revealed that very low levels of interleukin 5 (IL-5) (<15.86 pg/mL) in ascites and IL-15 (<12.40 pg/mL) in plasma were able to predict HCC onset with an accuracy of 81.8% and a sensitivity of 95.2%. Univariate analyses also showed that HCC, hepatitis B virus/hepatitis C virus infections, low levels of IL-5 and granulocyte-macrophage colony-stimulating factor in ascitic fluids, and high levels of eotaxin-1, hepatocyte growth factor and stromal-cell-derived factor 1α in plasma samples were factors potentially associated with a poor prognosis and decreased survival. Our results suggest a potential protective role of some immune modulators that may act in the peritoneal cavity to counteract disease progression leading to HCC development.

## 1. Introduction

Hepatocellular carcinoma (HCC) is the most common primary liver cancer, accounting for roughly 70% of cases, arising in the context of cirrhosis [1,2] principally in relation to chronic hepatitis B virus (HBV) or hepatitis C virus (HCV) infections [3]. HCC is generally diagnosed at a late stage, which significantly decreases the survival rate to less than 14% within five years, making it a major cause of cancer-related mortality [2,4].

Cirrhosis as chronic fibrotic liver disease represents the end-stage condition of a systemic inflammatory state, due to persistent production of soluble immunological factors such as interleukins, chemokines, growth factors, and matrix-degrading enzymes [5]. Their levels may vary according to different body compartments and require the engagement of different cell types including tumor cells, immune system cells, endothelial cells and mesenchymal stromal/stem cells. These last cell types can participate in each step of tumor development, and microenvironment stimuli could modify their immune status and activity, leading to the stimulation or suppression of the immune response [6,7]. The presence of inflammatory mediators and the imbalance of pro-inflammatory type 1 T-helper cells (Th1) vs. anti-inflammatory Th2 cytokines are key features that trigger the so-called non-resolving inflammation as a promoter of neoplastic transformation [8].

Many studies have documented increased levels of cytokines and chemokines in chronic liver diseases. In HCV patients, intrahepatic chemokines became elevated and correlated with the severity of disease; in alcoholic steatohepatitis, cytokines and chemokines increased further during acute decompensation, and the highest concentration of serum cytokines correlated with the highest rate of hospital mortality [4,9]. Many of these soluble factors are secreted or shed into the extracellular space and collected in the tumor or pre-tumor proximal fluids, including ascitic fluid. Ascitic fluid formation can occur both in malignant and benign disorders. Recently, a few chemokines were found in most cancerous ascitic fluids, and ovarian-cancer-related ascites represent the most studied ones [10,11]. Some significantly increased cytokines in ascitic fluids from HCC were found to induce the migration of hepatocellular cancer cell lines [10]. Indeed, the inflammatory microenvironment of ascitic fluid supports the transformation of hepatocytes, promoting their survival and favoring HCC development and metastasis [12].

Although many studies have monitored the serum levels of pro-inflammatory molecules that were found to be significantly increased in HCC patients compared to healthy controls, very little is known about cytokine and chemokine levels in pre-tumor or tumor proximal fluids and their comparison with serum levels [13]. The aim of this pilot study was to measure the amounts of 45 cytokines in the plasma and ascitic fluid of a cohort of cirrhotic patients with and without HBV/HCV infection and a cohort of HCC patients with and without HBV/HCV infection to evaluate the potential HCC predictive value of these molecules, their physiopathological role in disease progression, and their association with patient survival.

## 2. Results

### 2.1. Patients 

Ascites and blood samples were obtained from a total of 44 patients with liver cirrhosis, and 21 of them had developed HCC. Of the HCC patients, 10 had virus-associated HCC (1 with HBV, 1 with HBV/HCV, and 8 with HCV), whereas 2 were associated with monoclonal gammopathy of undetermined significance and the remaining with alcohol consumption. Of the 23 patients not affected by HCC, 10 had hepatitis B- or C-associated cirrhosis (9 with HCV and 1 with HBV), 2 were cryptogenetics and the remaining were associated with alcohol abuse. The demographic and clinical characteristics of the patients are reported in Table 1. 

### 2.2. Trend of Cytokines in the Two Body Compartments

**Figure 1:**
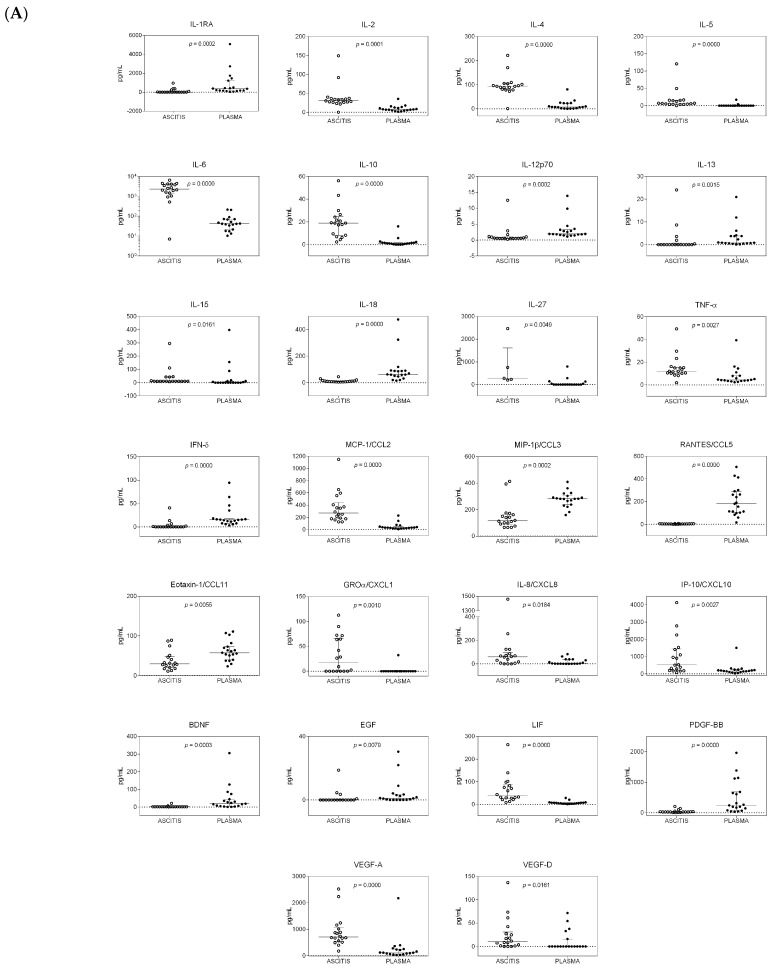

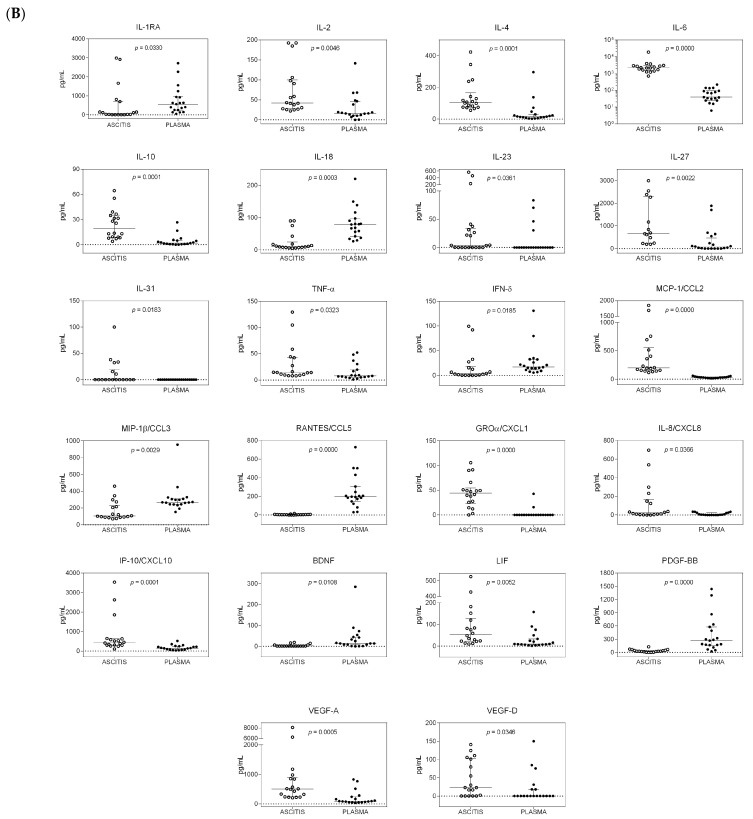
Distribution of cytokines in the ascitic and systemic compartments of patients with and without hepatocellular carcinoma (HCC). Dot plot graphs showing the distribution of the amounts of cytokines, measured in ascitic fluids and plasma samples and expressed as pg/mL, in patients with HCC (**A**) and in patients without HCC (**B**). Median and interquartile ranges are also reported. Only cytokines with a statistically significant differential distribution are shown. The statistical significance of the distribution of each cytokine between the two compartments is also reported and was calculated using the Kruskal–Wallis test, as data were not normally distributed. *p*-values were adjusted for multiple comparisons using the Benjamini–Hochberg (BH) method.

The comparison of cytokine levels in the ascites and plasma samples of all patients is shown in Figure 1. Only cytokines showing statistically significant (*p* < 0.05) different amounts in the two compartments in cirrhotic patients with or without HCC are reported. Twenty-six cytokines showed a statistically significant differential distribution in the two compartments, after the Benjamini–Hochberg (BH) readjustment for multiple comparisons in HCC patients (Figure 1A). Interleukin 1 receptor antagonist (IL-1Ra), IL-13, interferon γ (IFN-γ), brain-derived neurotrophic factor (BDNF), and epidermal growth factor (EGF) were never or sporadically found in ascitic samples, but they were detected in plasma samples. IL-5, IL-15, IL-27, growth-regulated oncogene α (GRO-α), and vascular endothelial growth factor D (VEGF-D) showed the opposite trend. Of the remaining 16 factors, IL-2, IL-4, IL-6, IL-10, tumor necrosis factor α (TNF-α), monocyte chemotactic protein 1 (MCP-1), IL-8, IFN-γ-induced protein 10 (IP-10), leukemia inhibitory factor (LIF), and VEGF-A were found to a higher extent in ascites, whereas IL-12p70, IL-18, macrophage inflammatory protein 1β (MIP-1β), regulated on activation, normal T-cell expressed and secreted (RANTES), eotaxin, and platelet-derived growth factor BB (PDGF-BB) were higher in plasma samples. 

In HCC patients, high levels of SDF-1α (>700 pg/mL) and hepatocyte growth factor (HGF) (>1200 pg/mL) were found in both body compartments. 

Twenty-two cytokines were found to have a statistically significant differential distribution in the systemic compared to the peritoneal compartment in cirrhotic patients without HCC (Figure 1B). Considering the median values, IL-23 and IL-31 were only found at low levels in a few ascitic samples, and GRO-α was consistently present in ascitic samples, but these three cytokines were never found in plasma samples. VEGF-D was mainly present in ascitic fluids and sporadically measured in plasma samples, whereas BDNF showed the opposite trend. The remaining 19 cytokines were consistently present in both body sites, but the levels of IL-2, IL-4, IL-5, IL-6, IL-10, IL-27, TNF-α, MCP-1, IL-8, IP-10, LIF, and VEGF-A were higher in ascitic fluids, whereas the levels of IL-1Ra, IL-18, IFN-γ, MIP-1β, RANTES, and PDGF-BB were higher in plasma samples. 

### 2.3. Trend of Cytokines in the Two Patient Cohorts

HCC patients showed lower amounts of released cytokines when compared to patients without HCC in both compartments, clearly indicating that, once the tumor develops, the pro-inflammatory and pro-tumorigenic condition eventually regresses. 

The comparison of the median levels of cytokines found in the ascites of both patient groups is shown in Table 2. After adjustment for multiple comparisons, a statistically significant difference in the levels of four cytokines was found. Specifically, IL-13, IL-17A, IFN-α, and IFN-γ were consistently detected in patients without HCC and were undetectable or sporadically measured in HCC patients.

In plasma samples, IL-5, IL-15 and IFN-α were detected in patients without HCC and rarely or never found in HCC patients; the amounts of IL-2 and granulocyte-macrophage colony-stimulating factor (GM-CSF) were significantly higher in patients without HCC. 

### 2.4. HCC Risk Prediction

A univariate logistic regression analysis was performed and the Youden cut-off value was estimated in order to detect cytokines in plasma and ascites, predicting HCC risk (Table S1). The analysis successfully identified 11 cytokines in each compartment. Interestingly, 7 of the 11 cytokines were selected in both compartments, specifically, 5 interleukins (IL-1β, IL-2, IL-5, IL-15, and IL-17A), IFN-α, and one growth factor (GM-CSF). Four cytokines were selected specifically for each compartment; in particular, 3 interleukins (IL-7, IL-13, and IL-23) and IFN-γ were selected in ascites, whereas IL-4, TNF-α, MIP-1α and LIF in plasma.

### 2.5. Classification Tree Predicting HCC

**Figure 2:**
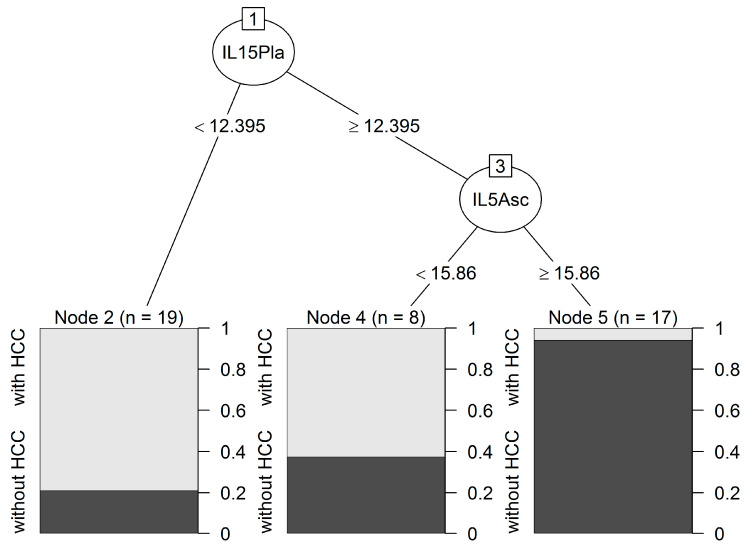
Classification tree predicting HCC. Two interleukins, IL-5 in ascites and IL-15 in plasma samples, were identified. Each path from the first node to a terminal node forms an interaction pattern and denotes a combination of predictors and their cut-off values. Cut-off values are indicated and were calculated using the Gini rule. This tree identified patients with these two cytokines with amounts above the two cut-off values (node 5) as the ones with the lowest risk of developing HCC and this prediction was accurate, as it correctly classified 16 of the 17 patients without HCC. The other two final nodes (2 and 4) corresponding to two interaction patterns classified patients with a high probability of developing HCC, and showed a lower accuracy (correctly classified 15 out of 19 and 5 out of 8 patients with HCC).

We used a classification tree method to identify interacting variables able to predict the likelihood of developing HCC. The analysis selected IL-5 in ascites and IL-15 in plasma samples; both interleukins were able to correctly predict the probability of HCC in a univariate logistic regression (Table S1). As shown in Figure 2, the first split identified 19 patients with plasmatic IL-15 < 12.40 pg/mL as HCC patients. Subjects with plasmatic IL-15 ≥ 12.40 pg/mL and IL-5 in ascites ≥ 15.86 pg/mL were classified as patients without HCC (node 5, 17 patients), whereas those with IL-5 < 15.86 pg/mL were identified as HCC patients (node 4, 8 patients). The overall accuracy of the classification was 81.8%, with a high sensitivity of 95.2% (only one false-negative patient) and a specificity of 69.6% (with seven false-positive patients). Patients with high values for both interleukins had the lowest risk of HCC (node 5 vs. node 2: OR 0.002; 95% CI 0–0.17; *p* < 0.001).

### 2.6. Trend of Cytokines in the Two Compartments in Subjects with or without HBV/HCV Infection

By comparing cytokine levels in the two compartments in patients without virus infection, we obtained 25 factors showing statistical significance (Table S2). By comparing cytokine levels in the two compartments in HBV/HCV-infected patients, 20 were significantly different (Table S3). Interestingly, the comparison of these two tables showed that 19 cytokines were in both lists, whereas IL-23, IL-31, TNF-α, IL-8, eotaxin, stem cell factor (SCF), and VEGF-D were specific to the group of patients with HBV/HCV infection.

Finally, the comparison between the two categories infected with HBV/HCV infection vs. patients without infection did not show any statistically significant results after correction for multiple tests (Tables S4 and S5).

### 2.7. Univariate Analyses for OS

**Figure 3:**
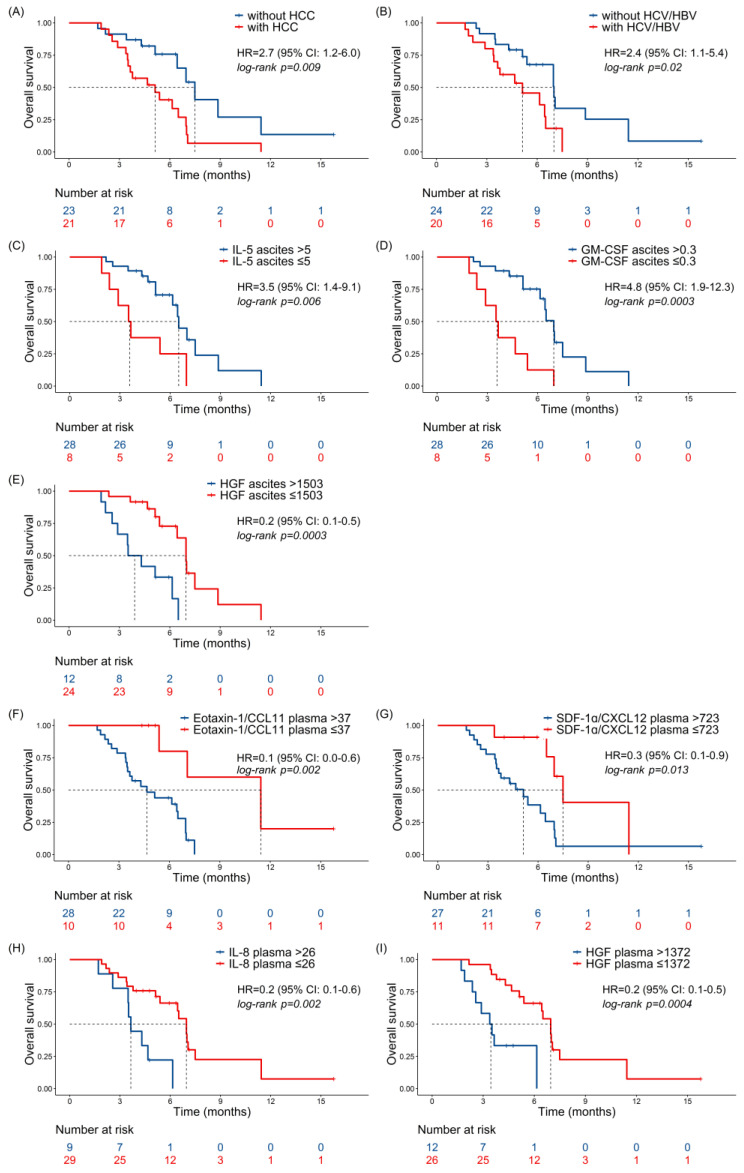
Prognostic significance of clinical parameters and cytokine levels. Kaplan–Meier curves for overall survival (OS) according to HCC state (**A**), HCV/HBV infection (**B**), and cytokine levels in ascitic fluids (**C**–**E**) and plasma samples (**F**–**I**) of the studied patients. Three patients with cirrhosis developed HCC during the study period and they were censored at the time of HCC assessment. Only significant variables are shown. Cytokine levels, expressed as pg/mL, were dichotomized according to optimal cut-points, estimated by maximizing the discriminative ability of the Cox model. HR and 95% CI are indicated, and the log-rank test was used to calculate the *p*-value, as indicated.

The presence of HCC or HBV/HCV infection was significantly associated with a shorter survival and a higher risk (Figure 3A,B, respectively). Low levels of IL-5 and GM-CSF in ascitic fluids were associated with a decreased survival and higher risk (Figure 3C,D). High amounts of HGF in ascitic fluids and in plasma samples (panels E and I), and eotaxin, IL-8, and SDF-1α in plasma samples (panels F–H, respectively) were significantly associated with a poor outcome. 

### 2.8. Classification Tree Predicting OS

**Figure 4:**
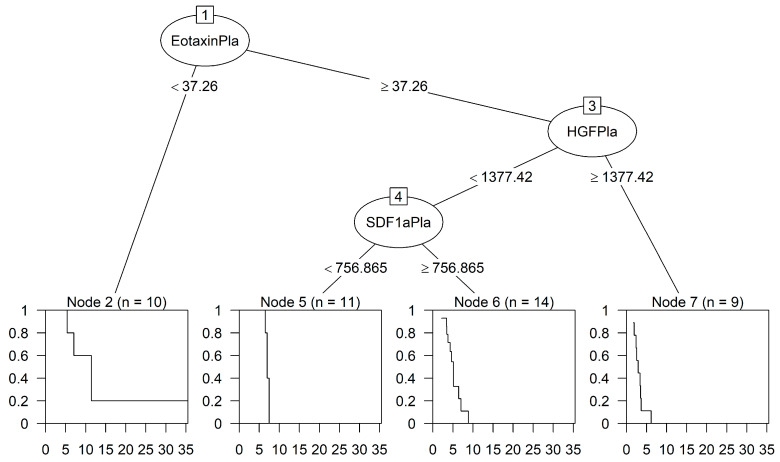
Classification tree predicting OS. The analyses selected two chemokines and one growth factor in plasma samples able to identify four interaction patterns leading to final nodes 2 and 5–7, as indicated. The cut-off values of these cytokines (pg/mL) are indicated, and the splitting rule used in generating the survival tree was based on the log-rank statistics. The curves inside the squares show the Kaplan–Meier estimated survival of each group of patients, and time is indicated in months.

Figure 4 shows a survival tree for the identification of interacting predictors of OS. Interestingly, two chemokines (eotaxin and SDF-1α) and one growth factor (HGF), relevant to HCC, detected in plasma (indicated as Pla) samples were selected as the most important predictors of survival. Node 1 at the top shows that eotaxin is the most important variable for the first split (node 2, eotaxin < 37.26 pg/mL; median OS, 348 days; 95% CI, 164–NA) followed by HGF and SDF-1α. The interaction pattern leading to node 5 (eotaxin ≥ 37.26; HGF < 1377.42; SDF-1α < 756.87) defined a higher risk (median OS, 212 days; 95% CI, 198–NA) compared to node 2, but the difference was not significant (node 5 vs. node 2: HR, 2.56; 95% CI 0.45–14.46; *p* = 0.286). The interaction patterns of node 6 (eotaxin ≥ 37.26; HGF < 1377.42; SDF-1α ≥ 756.87; median OS, 156 days; 95% CI, 106–196) and node 7 (eotaxin ≥ 37.26; HGF ≥ 1377.42; median OS, 88 days, 95% CI, 52–111) identified subgroups of patients with a statistically significantly lower survival and higher risk (node 6 vs. node 2: HR, 8.93; 95% CI, 1.94–41.16; *p* = 0.005; node 7 vs. node 2: HR, 45.71; 95% CI, 8.47–246.67; *p* < 0.0001).

## 3. Discussion

Cirrhosis-associated immune dysfunction derives from the impairment of the homeostatic role of the liver in the course of cirrhosis. This dysfunction, mainly represented by immunodeficiency and systemic inflammation, leads to the transition from an early predominantly “pro-inflammatory” phenotype of compensated ascitic cirrhosis to an “immunodeficient” phenotype, which is observed in decompensated liver cirrhosis with multi-organ failure [14,15,16,17]. Although this inflammatory syndrome is defined as a systemic event, it does not uniformly affect the whole organism but rather it acts in a compartmentalized manner [18,19,20]. In the course of cirrhosis, immune and non-immune cells, as well as a vast network of inflammatory mediators, i.e., cytokines, chemokines, and growth factors, are regulated at both the transcriptional and protein levels in a site-specific manner. In our pilot study, the quantitative analysis of 45 molecules defined a distinct cytokine profile for each of the two analyzed biological fluids, plasma and ascitic fluid, resulting in statistically significant differences between cirrhotic patients with or without HCC. Of note, patients with HCC showed drastically lower levels of these factors, in agreement with the immunosuppressive network established by the tumor [21,22]. 

Univariate analyses revealed that factors with a significant role in HCC risk prediction in the two biological compartments are two cytokines, IL-5 and IL-15, previously found to play a controversial role in cancer pathogenesis [23,24]. Our results suggest that their concomitant and exacerbated local production may be the result of spatially different pathophysiological events. In this context, previous studies showed that chemotactic factors, co-activators of macrophages, and effector natural killer (NK) cells may establish a particular phenotype only in the ascites of cirrhotic patients, further demonstrating that the immune response is compartmentalized during systemic inflammation in cirrhotic patients [18,20]. By using classification tree methods, our pilot study was able to identify a synergistic association between these cytokines, which proved to be excellent predictors of HCC development and good peri-diagnostic tools. It has to be pointed out that we used only one tree model, and further validation is needed. Nevertheless, IL-15 was shown to promote NK-mediated antitumor response [25,26]. Several preclinical models and clinical trials demonstrated the ability of IL-15 to counteract HCC-induced NK dysfunction, and these findings make it a promising cytokine for cancer immunotherapy [27,28,29,30,31,32]. The protective, antineoplastic role of this interleukin was confirmed in our study, as, although it was detected at very low levels in both biological compartments, it was present at higher levels in patients without HCC compared to HCC patients. Moreover, it was shown to predict HCC onset when its plasma levels were below the optimal threshold (12.40 pg/mL). As this cut-off value is derived from our patient population, further studies in independent patient cohorts are needed to confirm or readjust this threshold. 

The synergistic effect of the association between IL-5 and IL-15 strengthens the previously demonstrated protective role played by these two interleukins in the progression towards HCC. IL-5, which plays an important role in several allergic diseases, has been shown to exert its protective effect in cancer through the involvement of eosinophils, which act in concert with other immune cells in regulating the antineoplastic immunity [32]. By using a mouse model of allergic inflammation, Th2 cells, one of the major sources of IL-5, were shown to be involved in tumor rejection through the activation of innate immune cells, such as macrophages and eosinophils [33,34,35,36]. A decrease in eosinophil infiltration in addition to a reduction in IL-5 was observed after the depletion of NK cells [37,38]. However, the protective role of IL-5 was eosinophil-independent, and resulted in an increase in circulating neutrophils and monocytes [39]. This led to reduced mortality observed during polymicrobial sepsis, a condition mimicking the immune system imbalance of cirrhotic patients [40]. In our study, IL-5 was found to be a protective and prognostic factor in the ascitic compartment, a site in which its low levels correlated with a higher risk of HCC development and decreased survival. IL-5 levels higher than 15.86 pg/mL in the ascitic fluid not only have a favorable prognostic value, as a marker of longer survival, but also, in association with high plasma IL-15 levels (above 12.395 pg/mL), play a highly useful peri-diagnostic role in the management of the cirrhotic patient. In agreement with our findings, IL-5 was previously shown to be a marker of better survival in a retrospective study in which its plasma levels above 12 pg/mL correlated with higher survival in HCC patients treated with Sorafenib [41]. Moreover, IL-5 was recognized as a crucial mediator of immune checkpoint blockade response in breast cancer [36]. Although some studies have shown that IL-5 may promote cell metastasis in esophageal tumors in which it is produced and secreted [24,40,42], expression studies in epithelial ovarian cancer documented its presence in the peritoneal cavity, with significantly higher levels in benign versus malignant tumors [43], further confirming its protective role [44].

The peritoneal cavity represents an important microenvironment and an ideal immunological compartment for the study of the course of cirrhosis. In physiological conditions, the peritoneal cavity contains numerous mononuclear phagocytes and primary macrophages, but during inflammation, the expression of cytokines and chemokines can be triggered by other cells, such as activated mesothelial cells and fibroblastic stromal cells, which favor the recruitment of systemic leukocytes. In this context, the omentum plays an essential role in the peritoneal defense against bacterial infections, frequently found in ascites, and in the modulation of the local innate immune response of patients with cirrhosis [45,46,47]. Among the OS predictors identified in our pilot study, IL-5 and GM-CSF showed a favorable prognostic value, as their higher levels in ascitic fluids were found to be significantly associated with increased survival. These two factors, which share the same receptor beta subunit, exert pathophysiological effects at the site of inflammation and cancer where they are produced [48,49,50,51]. The IL-5 receptor is expressed on the neutrophils and monocytes of patients with sepsis [40]. In a preclinical model of sepsis, GM-CSF was shown to facilitate monocyte differentiation into macrophages, to induce the transition of rat mesenteric mesothelial cells into macrophage-like cells, and to support the antitumor activity of dendritic cells [52,53,54,55,56]. 

Mesothelial cells exert crucial functions in tissue homeostasis and play an immunoregulatory role not only through the secretion of various chemokines and cytokines but also through the expression of innate immunity receptors [57,58,59,60,61]. Many, in fact, of the soluble factors identified in this study and secreted in different amounts in the different compartments and patient cohorts, such as IL-15, MCP-1, RANTES, eotaxin-1, GRO-α, IL-8, IP-10, SDF-1α, GM-CSF, and HGF, are released by mesothelial cells. Among these, levels of eotaxin-1, IL-8, SDF-1α, and HGF above the cut-off value in plasma samples were associated with shorter survival in univariate analyses. Moreover, their association in plasma samples in a classification tree identified a set of patients with a poor prognosis. Although this is a preliminary finding, it is in line with the function exerted by these soluble factors. Indeed, eotaxin-1 is a chemokine involved in the pathogenesis of chronic liver disease and is recognized as a biomarker of liver inflammation, advanced fibrosis, and an adverse clinical course [62,63,64]. It can deflect the movement of basophils, fibroblasts, and neutrophils, and its expression appears to be induced by HGF through the mediation of Brg1 [65]. The significance of HGF is somehow controversial, as some authors have shown no differences in its serum levels in cirrhotic patients with and without HCC [66,67,68]. However, HGF was consistently found to be correlated with a poorer prognosis and shorter survival in HCC patients in several studies [69]. Moreover, its usefulness in diagnostics has been reliably proven, mainly in association with other biomarkers [70,71,72]. In our study, the plasma levels of HGF did not significantly differ between the two biological compartments but were significantly higher in patients with HCC. Finally, SDF-1α and HGF are known to be linked to the aggressiveness of several solid tumors and, in particular, HCC; they are inversely correlated with long-term survival and found to be implicated in metastatic spread [73,74,75,76].

In conclusion, our results show compartmentalized profiles of cytokines and highlight the fundamental role of some immune modulators acting in the peritoneal cavity. Our preliminary data, although obtained from a limited number of patients, identified cytokine associations, and specifically IL-5 and IL-15, potentially predictive of HCC onset in patients with decompensated liver cirrhosis. Moreover, eotaxin-1, HGF, and SDF-1 were shown to have a potential prognostic significance in HCC patients. Other cytokine combinations, such as IL-5 and GM-CSF, were found to be new potential therapeutic tools, and highlighted the ability of the subset of cytokine-releasing cells of the peritoneal cavity to counteract cirrhosis progression leading to HCC development. These preliminary findings need to be validated in independent and larger patient cohorts. 

## 4. Materials and Methods

### 4.1. Patients

Inclusion criteria were as follows: absence of clinical symptoms of active infection; normal erythrocyte sedimentation rate value; normal C-reactive protein value; absence of spontaneous bacterial peritonitis or bacteriascites.

Ascites were centrifuged, and supernatants were aliquoted and stored at −80 °C until use. Blood samples were processed, and plasma samples were aliquoted and stored at −20 °C until use.

### 4.2. Quantitative Analysis of Cytokines, Chemokines, and Growth Factors

To evaluate the profile and number of factors present in plasma samples and ascitic fluids, a Luminex xMAP approach was used (ProcartaPlex Human Cytokine/Chemokine/Growth Factor Panel 1 96 tests, Affymetrix eBioscience LTD, Hatfield, UK) for the multianalyte detection of 45 secreted proteins. This assay detects the following proteins: IL-1α, IL-1Ra, IL-1β, IL-2, IL-4, IL-5, IL-6, IL-7, IL-9, IL-10, IL-12p70, IL-13, IL-15, IL-17A, IL-18, IL-21, IL-22, IL-23, IL-27, IL-31, TNF-α, TNF-β, LIF, IFN-α, IFN-γ, MCP-1/C-C motif chemokine ligand 2 (CCL2), MIP-1α/CCL3, MIP-1β/CCL4, RANTES/CCL5, eotaxin/CCL11, GRO-α/C-X-C motif chemokine ligand 1 (CXCL1), IL-8/CXCL8, IP-10/CXCL10, SDF-1α/CXCL12, BDNF, nerve growth factor β, EGF, fibroblast growth factor 2 (FGF-2), GM-CSF, HGF, PDGF-BB, placental growth factor, SCF, VEGF-A, and VEGF-D.

Patient characteristics and cytokine distribution in the two compartments in cirrhotic patients with and without HCV/HBV infection are reported as median and interquartile range (Q1–Q3) or frequencies and percentages. 

To check the quality of the assay, observed reference curves of all cytokines in each plate were visually inspected and compared to the expected ones. The lowest detection thresholds of 2 of 45 cytokines (specifically IL-9 and FGF-2) were not available in each plate, suggesting systematic errors with these standards. Therefore, cytokines whose lowest threshold value was different in individual plates, and for which all sample values were <OR, were excluded from the analysis (specifically IL-9 and FGF-2). Values of IL-21 and TNF-β obtained from plasma samples were also excluded for the same reasons (all values <OR and lack of the lowest value of the standard curve), whereas the detected lowest values in the ascitic samples were considered reliable, as the most diluted standard was reproducible and detected in all plates. For IL-27, all values detected in the plasma samples were considered reliable as the observed standard curves were fine; however, as the standard curve lacked the most diluted sample in the plate with ascitic samples, only ascitic samples (the majority, however) showing values above the last point of the reference curve were included in the analysis. 

All samples were analyzed in duplicate and the median values were used for statistical analyses.

### 4.3. Statistical Analyses 

After checking that the data were not normally distributed using the Shapiro–Wilk normality test, a two-tailed Kruskal–Wallis test, with BH multiple test correction, was used to identify cytokines that were significantly different between compartments and HCC patient cohorts.

In order to distinguish low- and high-risk groups of patients, the cytokines were dichotomized with the optimal threshold corresponding to the higher Youden’s statistic and included in univariate logistic regression models predicting HCC risk. Logistic regression results are shown as OR with 95% confidence intervals.

Overall survival (OS) was the clinical outcome and was calculated from the date of blood/ascites sample collection to the date of death. Patients who did not develop an event during the study period were censored at the date of the last observation. Patients with cirrhosis who developed HCC during the study period were censored at the time of HCC assessment. The cytokines were analyzed for association with overall survival as categorical variables according to high and low levels. Optimal cut-points were estimated by maximizing the discriminative ability of the Cox model.

We conducted a classification tree for HCC risk and a survival regression tree for OS to determine the diagnostic/prognostic impact of the cytokines measured in the two compartments, using the “rpart” R package (version 4.1.19). The tree model recursively separates the covariate space into groups containing observations of homogeneous response values, called “nodes”. This procedure is applied until there is no longer a statistically significant split.

Tree results are shown as an odds ratio (OR) and hazard ratio (HR) with a 95% confidence interval obtained from a logistic model and a Cox proportional hazards regression model using, as a covariate, a new variable based on the final nodes. The median OS for each terminal node of the survival tree was estimated using the Kaplan–Meier method and reported with a 95% confidence interval.

All statistical tests were two-sided and a *p*-value < 0.05 was considered statistically significant. Statistical analyses were performed using RStudio version 2022.07.2+576 (RStudio: Integrated Development for R. RStudio Inc., v. 2022.07.2+576, Boston, MA, USA).

## Figures and Tables

**Table 1:** Summary of demographic and clinical characteristics of patients included in the study.

		with HCC ^1^ with Virus	with HCC without Virus	without HCC with Virus	without HCC without Virus	Total
Total	N	10	11	10	13	44
Age	Median (Q1, Q3)	59 (56, 72)	63 (55, 65)	63 (53.50, 74)	66 (54, 69)	63 (54, 69.5)
Gender	Female	3 (30.0%)	4 (36.4%)	5 (50.0%)	7 (53.8%)	19 (43.2%)
	Male	7 (70.0%)	7 (63.6%)	5 (50.0%)	6 (46.2%)	25 (56.8%)
HBsAg ^2^	Positive	2 (20.0%)	0 (0.0%)			2 (9.5%)
	Negative	8 (80.0%)	11 (100.0%)			19 (90.5%)
Tumor encapsulation	Yes	1 (10.0%)	1 (9.1%)			2 (9.5%)
Diameter (cm)	≤5	6 (60.0%)	7 (63.6%)			13 (61.9%)
	>5	4 (40.0%)	4 (36.4%)			8 (38.1%)
Tumor multiplicity	Multiple	8 (80.0%)	9 (81.8%)			17 (81.0%)
	Solitary	2 (20.0%)	2 (18.2%)			4 (19.0%)
Histological grade	Poorly differentiated	5 (50.0%)	4 (36.4%)			9 (42.9%)
	Moderately differentiated	3 (30.0%)	4 (36.4%)			7 (33.3%)
	Well differentiated	2 (20.0%)	3 (27.3%)			5 (23.8%)
Alpha-fetoprotein	Median (Q1, Q3)	34.9 (8.2, 904.2)	36.0 (8.4, 183.0)			36.00 (6.3, 344.0)
Microvascular invasion	Yes	5 (50.0%)	6 (54.5%)			11 (52.4%)
AJCC ^3^ stage	I	2 (20.0%)	2 (18.2%)			4 (19.0%)
	II	0 (0.0%)	1 (9.1%)			1 (4.8%)
	III	3 (30.0%)	2 (18.2%)			5 (23.8%)
	IV	5 (50.0%)	6 (54.5%)			11 (52.4%)
T ^4^	T1	2 (20.0%)	2 (18.2%)			4 (19.0%)
	T2	2 (20.0%)	2 (18.2%)			4 (19.0%)
	T3	6 (60.0%)	6 (54.6%)			12 (27.3%)
	T4	0 (0.0%)	1 (9.1%)			1 (4.8%)
N	N0	5 (50.0%)	3 (27.3%)			8 (38.1%)
	N1	4 (40.0%)	6 (54.5%)			10 (47.6%)
	NX	1 (10.0%)	2 (18.2%)			3 (14.3%)
M	M0	8 (80.0%)	9 (81.8%)			17 (81.0%)
	M1	2 (20.0%)	2 (18.2%)			4 (19.0%)

^1^ Hepatocellular carcinoma; ^2^ Hepatitis B surface antigen; ^3^ American Joint Committee on Cancer; ^4^ The TNM staging system describes the size of the primary tumor (T), metastases in regional lymph nodes (N), and distant sites (M).

**Table 2:** Cytokine amounts in the ascitic and systemic compartments of patients with and without hepatocellular carcinoma (HCC).

	without HCC	with HCC	Total	*p*-Value
Total	23	21	44	
Ascites				
IL-13	4.12 (0.60, 9.40)	0.00 (0.00, 0.26)	0.38 (0.00, 5.75)	0.041
IL-17A	15.95 (4.43, 41.40)	0.00 (0.00, 3.70)	4.44 (0.00, 20.36)	0.041
IFN-α	1.90 (0.60, 5.99)	0.00 (0.00, 0.86)	0.69 (0.00, 2.84)	0.041
IFN-γ	3.52 (0.47, 14.74)	0.00 (0.00, 1.56)	0.90 (0.00, 6.88)	0.041
Plasma				
IL-2	16.12 (11.21, 42.38)	7.44 (5.18, 11.65)	11.54 (6.71, 17.80)	0.041
IL-5	1.41 (0.00, 27.71)	0.00 (0.00, 0.00)	0.00 (0.00, 6.94)	0.041
IL-15	39.57 (15.66, 93.03)	0.00 (0.00, 11.56)	14.04 (0.00, 50.77)	0.041
IFN-α	0.37 (0.04, 1.61)	0.00 (0.00, 0.03)	0.04 (0.00, 0.85)	0.041
GM-CSF	37.85 (11.30, 78.72)	2.60 (0.00, 17.70)	14.37 (0.00, 49.10)	0.041

Only cytokines with a statistically significant differential distribution are shown. Median levels, expressed as pg/mL, and interquartile ranges are reported. The Kruskal–Wallis test was used for comparisons between patients with and without HCC, as data were not normally distributed. *p*-values were adjusted for multiple comparisons using the BH method.

## Data Availability

All data, if not already included in the manuscript, are available from the corresponding author on reasonable request.

## Supplementary Materials

The supporting information can be downloaded at: https://www.mdpi.com/article/10.3390/ijms241713432/s1.
