# Supplementary material for: Studies on the Role of Compartmentalized Profiles of Cytokines in the Risk of Hepatocellular Carcinoma
Source: Int J Mol Sci. 2023 Aug 30;24(17):13432. doi: 10.3390/ijms241713432 (PMC10563083; doi:10.3390/ijms241713432)
Supplement: Supplementary file 1 [file ijms-24-13432-s001.zip › ijms-2519995-supplementary.pdf]

Table S1. Univariate logistic regression predicting hepatocellular carcinoma (HCC) risk

	Youden cutoff	without HCC	with HCC	OR (95%CI)	*P* value*
**Ascites**					
**IL-1β**	0	6	14	7 (1.59, 30.8)	*0.046*
	> 0	12	4		
**IL-2**	< 36.64	6	15	10 (2.06, 48.56)	*0.032*
	≥ 36.64	12	3		
**IL-5**	< 15.68	6	15	10 (2.06, 48.56)	*0.032*
	≥ 15.68	12	3		
**IL-7**	< 0.63	4	13	9.1 (2, 41.45)	*0.032*
	≥ 0.63	14	5		
**IL-13**	< 0.35	4	14	12.25 (2.54, 58.97)	*0.028*
	≥ 0.35	14	4		
**IL-15**	< 10.64	4	12	7 (1.59, 30.8)	*0.046*
	≥ 10.64	14	6		
**IL-17A**	< 0.56	3	12	10 (2.06, 48.56)	*0.032*
	≥ 0.56	15	6		
**IL-23**	0	8	14	7.58 (1.65, 34.9)	*0.046*
	> 0	13	3		
**IFN-α**	< 0.06	3	13	13 (2.59, 65.2)	*0.028*
	≥ 0.06	15	5		
**IFN-γ**	0	2	12	16 (2.73, 93.62)	*0.028*
	> 0	16	6		
**GM-CSF**	< 20.68	5	13	6.76 (1.57, 29.07)	*0.046*
	≥ 20.68	13	5		
**Plasma**					
**IL-1β**	< 1.33	3	11	7.33 (1.58, 33.97)	*0.046*
	≥ 1.33	16	8		
**IL-2**	< 8.39	3	12	9.14 (1.95, 42.9)	*0.032*
	≥ 8.39	16	7		
**IL-4**	< 10.34	5	13	6.07 (1.49, 24.76)	*0.046*
	≥ 10.34	14	6		
**IL-5**	0	8	17	11.69 (2.08, 65.61)	*0.032*
	> 0	11	2		
**IL-15**	< 11.56	3	15	20 (3.82, 104.6)	*0.008*
	≥ 11.56	16	4		
**IL-17A**	0	3	12	9.14 (1.95, 42.9)	*0.032*
	> 0	16	7		
**TNF-α**	< 4.72	3	11	7.33 (1.58, 33.97)	*0.046*
	≥ 4.72	16	8		
**IFN-α**	0	4	14	10.5 (2.34, 47.2)	*0.028*
	> 0	15	5		
**MIP-1α/CCL3**	< 13.94	3	11	7.33 (1.58, 33.97)	*0.046*
	≥ 13.94	16	8		
**GM-CSF**	< 6.94	2	12	14.57 (2.57, 82.73)	*0.028*
	≥ 6.94	17	7		
**LIF**	< 7.76	6	14	6.07 (1.49, 24.76)	*0.046*
	≥ 7.76	13	5		

*The Benjamini-Hochberg multiple test correction was applied.

Table S2. Cytokine distribution in the two compartments in 24 cirrhotic patients without HCV/HBV infection

	Ascites	Plasma	Total	*P value**
**Interleukins, TNF and IFN**				
**IL-1Ra**	25.34 (0, 147.51)	364.25 (159.52, 696.16)	147.51 (25.34, 425.09)	*0.001*
**IL-2**	34.64 (27.69, 58.69)	11.54 (6.83, 16.41)	27 (12.19, 43.17)	*0.0001*
**IL-4**	96.62 (87.72, 118.13)	8.69 (4.78, 22.83)	66.32 (9.89, 101.27)	*0.0000*
**IL-5**	16.04 (6.93, 34.61)	0 (0, 4.16)	6.93 (0, 19.44)	*0.002*
**IL-6**	2403.61 (1656.98, 4129.58)	41.51 (22.46, 71.31)	517.6 (41.89, 2403.61)	*0.0000*
**IL-10**	21.53 (9.54, 31.56)	1.31 (0.89, 1.98)	7.21 (1.45, 24.28)	*0.0000*
**IL-12p70**	0.69 (0.56, 1.86)	2.08 (1.64, 3.16)	1.64 (0.69, 2.89)	*0.007*
**IL-18**	8.12 (6.79, 12.69)	60.37 (47.53, 90.71)	26.71 (8.12, 60.70)	*0.0000*
**IL-23**	0 (0, 26.50)	0 (0, 0)	0 (0, 2.70)	*0.020*
**IL-27**	722.36 (623.47, 1912.30)	12195 (0, 41.16)	42.84 (0.09, 682.37)	*0.0004*
**IL-31**	0 (0, 10.72)	0 (0, 0)	0 (0, 0)	*0.019*
**TNF-α**	13.41 (10.61, 16.16)	5.55 (3.96, 9.81)	10.61 (5.91, 15.23)	*0.001*
**IFN-γ**	0.85 (0, 7.26)	13.83 (8.92, 15.97)	7.71 (0.85, 14.63)	*0.001*
**Chemokines**				
**MCP1/CCL2**	227.41 (176.95, 514.93)	29.63 (20.65, 39.27)	124.75 (29.91, 229.76)	*0.0000*
**MIP-1β/CCL4**	108.37 (87.92, 205.86)	281.03 (241.52, 325.55)	232.7 (108.37, 291.50)	*0.001*
**RANTES/CCL5**	4.28 (3.01, 5.42)	192.24 (109.27, 260.56)	15.93 (4.28, 191.87)	*0.0000*
**GRO-α/CXCL1**	39.84 (9.14, 65.72)	0 (0, 0)	0 (0, 39.84)	*0.0000*
**IL-8/CXCL8**	37.79 (8.72, 124.11)	7.81 (0.73, 27.43)	24.54 (3.05, 62.73)	*0.011*
**IP-10/CXCL10**	508.52 (322.78, 1101.51)	142.92 (87.49, 245.25)	261.95 (143.61, 520.72)	*0.0000*
**Growth factors**				
**BDNF**	1.72 (1.72, 1.72)	14.87 (2.55, 37.20)	1.94 (1.72, 16.35)	*0.003*
**LIF**	58.83 (28.08, 83.19)	7.45 (4.84, 11.37)	24.42 (7.70, 70.94)	*0.0001*
**PDGF-BB**	27.24 (14.20, 40.75)	233.85 (102.06, 614.94)	48.45 (27.24, 198.34)	*0.0000*
**SCF**	10.81 (7.19, 19.59)	23085 (15.83, 46.81)	16.15 (9.57, 30.63)	*0.014*
**VEGF-A**	659.32 (432.56, 878.48)	111.37 (59.76, 254.95)	289.96 (112.11, 680.02)	*0.0002*
**VEGF-D**	16.67 (2.28, 42.59)	0 (0, 4.53)	2.28 (0, 29.97)	*0.011*

*The Kruskal—Wallis test was performed with Benjamini—Hochberg multiple test correction. Median levels, expressed as pg/ml, and interquartile range are reported.

Table S3. Cytokine distribution in the two compartments in 20 cirrhotic patients with HCV/HBV infection.

Cytokines	Ascites	Plasma	Total	*P value**
**Interleukins, TNF and IFN**				
**IL-1Ra**	25.34 (0, 306.74)	497.31 (199.49, 1153.77)	249.96 (31.82, 814.23)	*0.017*
**IL-2**	32.57 (23.96, 40.71)	11.54 (6.71, 18.68)	22.2 (10.22, 37.63)	*0.012*
**IL-4**	93.73 (79.69, 106.34)	12.69 (6.79, 23.77)	71.6 (10.79, 93.73)	*0.001*
**IL-5**	8.52 (5.40, 17.29)	0 (0, 6.94)	5.35 (0, 15.56)	*0.017*
**IL-6**	2146.52 (1532.63, 2699.08)	41.13 (31.63, 86.17)	93.14 (38.07, 2124.44)	*0.0002*
**IL-10**	13.76 (8.26, 24.40)	1.33 (0.72, 4.78)	5.72 (1.11, 16.09)	*0.001*
**IL-12p70**	0.84 (0.57, 1.42)	3.17 (1.96, 4.90)	1.93 (1.01, 3.97)	*0.010*
**IL-18**	12.12 (7.85, 19.73)	80075 (50.73, 90.32)	42.45 (12.94, 85.71)	*0.001*
**IL-27**	238.15 (225.15, 1175.00)	89.15 (0, 203.32)	180795 (0, 279.01)	*0.012*
**IFN-γ**	1.41 (0, 5.43)	19.5 (15.49, 32.37)	15.44 (1.76, 27.25)	*0.004*
**Chemokines**				
**MCP-1/CCL2**	248.86 (158.08, 380.58)	31.37 (24.25, 39.91)	67.27 (1.31, 202.86)	*0.0000*
**MIP-1β/CCL4**	112.72 (94.65, 134.85)	275815 (261.72, 298.03)	245.44 (118.51, 285.47)	*0.001*
**RANTES/CCL5**	3.04 (2.24, 3.46)	195.27 (127.49, 284.06)	32.8 (3.21, 205.53)	*0.0000*
**Eotaxin/CCL1**	30.93 (22.38, 45.51)	59735 (50.74, 71.14)	50.7 (30.93, 64.40)	*0.016*
**GRO-α/CXCL1**	26.35 (2.58, 49.40)	(0, 0)	(0, 32.33)	*0.001*
**IP-10/CXCL10**	411.95 (232.72, 930.84)	181.86 (111.24, 215.92)	208.2 (149.97, 411.95)	*0.011*
**Growth factors**				
**BDNF**	1.72 (1.72, 1.72)	21.99 (9.91, 51.72)	6.76 (1.72, 25.34)	*0.001*
**LIF**	33.27 (21.45, 120.99)	7915 (5.65, 18.06)	20.72 (7.76, 33.27)	*0.001*
**PDGF-BB**	27.24 (24.60, 84.47)	255775 (167.83, 631.07)	138.57 (27.24, 267.67)	*0.0002*
**VEGF-A**	662.97 (450.37, 938.77)	95.39 (81.52, 201.48)	233.79 (94.66, 662.97)	*0.0002*

*The Kruskal–Wallis test was performed with Benjamini–Hochberg multiple test correction. Median levels, expressed as pg/ml, and interquartile range are reported.

Table S4. Cytokine distribution in the ascites of cirrhotic patients with and without HCV/HBV infection.

	without HCV/HBV infection	with HCV/HBV infection	Total	*P value**
**Interleukins, TNF and IFN**				
**IL-1Ra**	25.34 (0, 147.51)	25.34 (0, 306.74)	25.34 (0, 175.24)	*0.9334*
**IL-2**	34.64 (27.69, 58.69)	32.57 (23.96, 40.71)	34.365 (26.90, 56.48)	*0.9334*
**IL-4**	96.62 (87.72, 118.13)	93.73 (79.69, 106.34)	96.215 (80.34, 112.48)	*0.9334*
**IL-5**	16.04 (6.93, 34.61)	8.52 (5.40, 17.29)	13.565 (5.43, 29.19)	*0.9334*
**IL-6**	2403.61 (1656.98, 4129.58)	2146.52 (1532.63, 2699.08)	2204.17 (1569.3, 3520.4)	*0.9334*
**IL-10**	21.53 (9.54, 31.56)	13.76 (8.26, 24.40)	18.93 (8.88, 30.37)	*0.9334*
**IL-12p70**	0.69 (0.56, 1.86)	0.84 (0.57, 1.42)	0.78 (0.55, 1.75)	*0.9334*
**IL-18**	8.12 (6.79, 12.69)	12.12 (7.85, 19.73)	9.44 (7.10, 16.50)	*0.9334*
**IL-27**	722.36 (623.47, 1912.30)	238.15 (225.15, 1175.00)	657.6 (234.59, 1722.28)	*0.9334*
**IFN-γ**	0.85 (0, 7.26)	1.41 (0, 5.43)	0.905 (0, 6.88)	*0.9334*
**Chemokines**				
**MCP-1/CCL2**	227.41 (176.95, 514.93)	248.86 (158.08, 380.58)	238.135 (169.39, 433.07)	*0.9334*
**MIP-1β/CCL4**	108.37 (87.92, 205.86)	112.72 (94.65, 134.85)	111.795 (90.99, 172.64)	*0.9334*
**RANTES/CCL5**	4.28 (3.01, 5.42)	3.04 (2.24, 3.46)	3.305 (2.77, 4.72)	*0.9334*
**Eotaxin/CCL1**	31.91 (28.16, 40.52)	30.93 (22.38, 45.51)	31.725 (26.37, 45.09)	*0.9334*
**GRO-α/CXCL1**	39.84 (9.14, 65.72)	26.35 (2.58, 49.40)	39.03 (2.69, 63.90)	*0.9334*
**IP-10/CXCL10**	508.52 (322.78, 1101.51)	411.95 (232.72, 930.84)	477.695 (294.40, 992.48)	*0.9334*
**Growth factors**				
**BDNF**	1.72 (1.72, 1.72)	1.72 (1.72, 1.72)	1.72 (1.72, 1.72)	*0.9334*
**LIF**	58.83 (28.08, 83.19)	33.27 (21.45, 120.99)	45.995 (24.11, 87.43)	*0.9334*
**PDGF-BB**	27.24 (14.20, 40.75)	27.24 (24.60, 84.47)	27.24 (19.08, 44.35)	*0.9334*
**VEGF-A**	659.32 (432.56, 878.48)	662.97 (450.37, 938.77)	661.145 (422.79, 904.68)	*0.9334*

*The Kruskal–Wallis test was performed with Benjamini–Hochberg multiple test correction. Median levels, expressed as pg/ml, and interquartile range are reported

Table S5. Cytokine distribution in the plasma compartment of cirrhotic patients with and without HCV/HBV infection.

	**without HCV/HBV infection**	**with HCV/HBV infection**	**Total**	***P value****
**Interleukins, TNF and IFN**				
**IL-1Ra**	364.25 (159.52, 696.16)	497.31 (199.49, 1153.77)	403.47 (176.18, 938.86)	*0.9334*
**IL-2**	11.54 (6.83, 16.41)	11.54 (6.71, 18.68)	11.54 (6.71, 17.80)	*0.9334*
**IL-4**	8.69 (4.78, 22.83)	12.69 (6.792, 23.77)	11.32 (5.54, 23.30)	*0.9334*
**IL-5**	0 (0, 4.16)	0 (0, 6.94)	0 (0, 6.94)	*0.9334*
**IL-6**	41.51 (22.47, 71.31)	41.13 (31.63, 86.17)	41.51 (25.93, 75.48)	*0.9334*
**IL-10**	1.31 (0.89, 1.98)	1.33 (0.72, 4.78)	1.31 (0.72, 2.74)	*0.9334*
**IL-12p70**	2.08 (1.64, 3.16)	3.17 (1.96, 4.90)	2.455 (1.81, 3.88)	*0.9334*
**IL-18**	60.37 (47.53, 90.71)	80.075 (50.73, 90.32)	68.82 (48.18, 90.65)	*0.9334*
**IL-27**	12.195 (0, 41.16)	89.15 (0, 203.32)	21.02 (0, 137.31)	*0.9334*
**IFN-γ**	13.83 (8.92, 15.97)	19.5 (15.49, 32.37)	15.54 (11.20, 24.93)	*0.9334*
**Chemokines**				
**MCP-1/CCL2**	29.635 (20.65, 39.27)	31.37 (24.25, 39.91)	31.195 (21.20, 39.91)	*0.9334*
**MIP-1β/CCL4**	281.03 (241.52, 325.55)	275.815 (261.72, 298.03)	280.92 (245.0, 305.01)	*0.9334*
**RANTES/CCL5**	192.245 (109.27, 260.56)	195.275 (127.49, 284.06)	192.245 (113.68, 284.06)	*0.9334*
**Eotaxin/CCL1**	43.105 (33.66, 58.95)	59.735 (50.74, 71.14)	53.475 (37.13, 68.22)	*0.9334*
**GRO-α/CXCL1**	0 (0, 0)	0 (0, 0)	0 (0, 0)	*0.9334*
**IP-10/CXCL10**	142.92 (87.49, 245.25)	181.865 (111.24, 215.92)	163.805 (96.80, 238.93)	*0.9334*
**Growth factors**				
**BDNF**	14.875 (2.5, 37.20)	21.99 (9.91, 51.72)	16.42 (6.85, 44.0)	*0.9334*
**LIF**	7.45 (4.84, 11.37)	7.915 (5.65, 18.06)	7.73 (5.21, 14.78)	*0.9334*
**PDGF-BB**	233.85 (102.06, 614.94)	255.775 (167.83, 631.07)	255.775 (150.68, 628.51)	*0.9334*
**VEGF-A**	111.37 (59.76, 254.95)	95.395 (81.52, 201.48)	103.38 (63.46, 240.5963)	*0.9334*

*The Kruskal–Wallis test was performed with Benjamini–Hochberg multiple test correction. Median levels, expressed as pg/ml, and interquartile range are reported
